# Involvement of cell cycle and ion transferring in the salt stress responses of alfalfa varieties at different development stages

**DOI:** 10.1186/s12870-023-04335-3

**Published:** 2023-06-27

**Authors:** YanLing Yin, ShuGao Fan, Shuang Li, Erick Amombo, JinMin Fu

**Affiliations:** Coastal Salinity Tolerant Grass Engineering and Technology Research Center, Ludong University, Yantai, Shandong People’s Republic of China

**Keywords:** Salt tolerance, Primary root, Cell cycle, Young seedling, Na^+^ distribution, *Medicago sativa*

## Abstract

**Background:**

Alfalfa (*Medicago sativa*) is the worldwide major feed crop for livestock. However, forage quality and productivity are reduced by salt stress, which is a common issue in alfalfa-growing regions. The relative salt tolerance is changed during plant life cycle. This research aimed to investigate the relative salt tolerance and the underlying mechanisms of two alfalfa varieties at different developmental stages.

**Results:**

Two alfalfa varieties, "Zhongmu No.1 (ZM1)" and "D4V", with varying salt tolerance, were subjected to salt stress (0, 100, 150 mM NaCl). When the germinated seeds were exposed to salt stress, D4V exhibited enhanced primary root growth compared to ZM1 due to the maintenance of meristem size, sustained or increased expression of cell cycle-related genes, greater activity of antioxidant enzymes and higher level of IAA. These findings indicated that D4V was more tolerant than ZM1 at early developmental stage. However, when young seedlings were exposed to salt stress, ZM1 displayed a lighter wilted phenotype and leaf cell death, higher biomass and nutritional quality, lower relative electrolytic leakage (EL) and malondialdehyde (MDA) concentration. In addition, ZM1 obtained a greater antioxidant capacity in leaves, indicated by less accumulation of hydrogen peroxide (H_2_O_2_) and higher activity of antioxidant enzymes. Further ionic tissue-distribution analysis identified that ZM1 accumulated less Na^+^ and more K^+^ in leaves and stems, resulting in lower Na^+^/K^+^ ratio, because of possessing higher expression of ion transporters and sensitivity of stomata closure. Therefore, the relative salt tolerance of ZM1 and D4V was reversed at young seedling stages, with the young seedlings of the former being more salt-tolerant.

**Conclusion:**

Our data revealed the changes of relative order of salt tolerance between alfalfa varieties as they develop. Meristem activity in primary root tips and ion transferring at young seedling stages were underlying mechanisms that resulted in differences in salt tolerance at different developmental stages.

**Supplementary Information:**

The online version contains supplementary material available at 10.1186/s12870-023-04335-3.

## Background

Soil salinity is becoming one of the most common threats to plant life and agricultural productivity. As a result of irrigation, climate change and natural processes, there are 800 million hectares of salt-alkalized soil in the world, one-tenth of which is cultivated land [1]. Salt stress causes physiological, metabolic, morphologic and molecular adaptations that impede growth and development, disrupt metabolism, and even cause plant death [2, 3]. Plant responses to salt stress varies according to the developmental stage, severity and duration of stress, and the salt tolerance of plant variety [4].

Roots anchor plants and absorb water and nutrients as subterranean tissues. They are also the first organs to perceive salt stress, especially the primary roots during the early embryonic stage [5]. After germination, the successful formation of primary root depends on the activation of cell division in the root apical meristem zone and cell size expansion in the elongation zone [6]. The meristem zone is particularly sensitive to salt stress in the rhizosphere, and it is responsible for the preliminary salt sensing [7]. Several investigations have convincingly demonstrated that salt stress can restrict the growth of primary roots by decreasing the number of cells in the meristem zone, as well as reducing cell division activity and rates [8–10]. The cell division is a successive cycle with two major checkpoints, G1 to S and G2 to M transition. Generally, cyclin and cyclin-dependent kinase (CDK) drive the progressions through these boundaries [11]. Salt stress was reported to inhibit cell cycle related genes at both transcriptional and posttranslational levels [10, 12]. The cell cycle succession under salt stress was critical for meristematic activity and primary root fostering.

After photomorphogenesis, the salt stress reaction is not restricted to the root but affects the entire plant. Wilt, bleaching and defoliation are all symptoms of salt stress, which is caused by osmotic stress and ion toxicity [13]. Osmotic stress is an early reaction caused by an excess of Na^+^ in the rhizosphere and limits water accessibility [14]. Once the Na^+^ is absorbed by the root, it undergoes upward transportation in xylem sap and finally accumulates in the leaf. Toxic dose of Na^+^ in the cytoplasm disrupts ionic homeostasis by, for example, limiting K^+^ absorption, which is necessary for the maintenance of enzyme activity and cell metabolisms [13, 15]. To maintain ion homeostasis, plants must limit Na^+^ buildup while increase K^+^ absorption to achieve an optimal cytoplastic ratio of Na^+^ to K^+^ [3]. Emerging evidence suggests that salt-tolerant plants are benefited from techniques of reducing Na^+^ allocation and maintaining low Na^+^/K^+^ levels in functioning leaves [7, 16, 17]. This pattern is achieved by lowering Na^+^ loading in xylem sap [18], retrieving Na^+^ from shoots [19], or restricting Na^+^ absorption by roots [19, 20]. These pathways are mediated by a group of ion transporters, including the salt-overly-sensitive1 (SOS1) [21], Na^+^/H^+^ exchanger (NHX), and high-affinity Na^+^/K^+^-permeable transporter (HKT) [22].

Alfalfa (*Medicago sativa*) is known as the "king of herbage" because of its high yield, rich crude protein content and digestible minerals [23]. Alfalfa is native to Central Asia, where salinity soil is common [24]. However, alfalfa may be planted on terrain with neutral or moderate salinity, but extreme salinization threatens its survival [24, 25]. Improving salt tolerance via breeding will lessen the threat of salt stress on alfalfa and contribute to the sustainability of pastoralism. Previous studies found that salt tolerance differs amongst alfalfa types in terms of physiological response, growth adaptability, proteomic alterations and genetic diversity [8, 26–28]. However, resistance at one developmental stage does not imply resistance at the next [29, 30]. Salt tolerance in one genotype may vary at different developmental stages, and these changes may differ among genotypes, resulting in variations in the relative order of salt tolerance at different developmental stages. Elucidating the specific physiological and molecular responses at different developmental stage is essential for identifying the most salt-tolerant variety at each stage. Additionally, such a study is fundamental for understanding the underlying mechanisms of salt tolerance throughout the life cycle. In this work, we looked at how two alfalfa varieties, ZM1 and D4V, responded to salt stress with a particular emphasis on primary root elongation at early developmental stage and ion tissue-distribution at young seedling stage.

## Results

### Primary root growth at the early developing stage under salt stress

The seed germination response to gradient increasing salt stress were estimated firstly. As shown in Fig. S1A, seed germination of all varieties was promoted by a low concentration of NaCl (25 mM), and NaCl at 50 mM exerted a negligible effect on seed germination. However, seed germination of most varieties was inhibited under 100 mM NaCl, with the exception of D4V and WL-SQT. D4V was found to be the most salt tolerant compared to other varieties under 150 mM NaCl. The primary root growth of all varieties was severely reduced by NaCl at 150 mM, with D4V and WL-SQT showing the longest primary roots, compared to other varieties (Fig. S1B and C). Based on the results of primary root growth and seed germination, D4V and ZM1 were chosen with different salt tolerance at the early developmental stage.

**Fig. 1:**
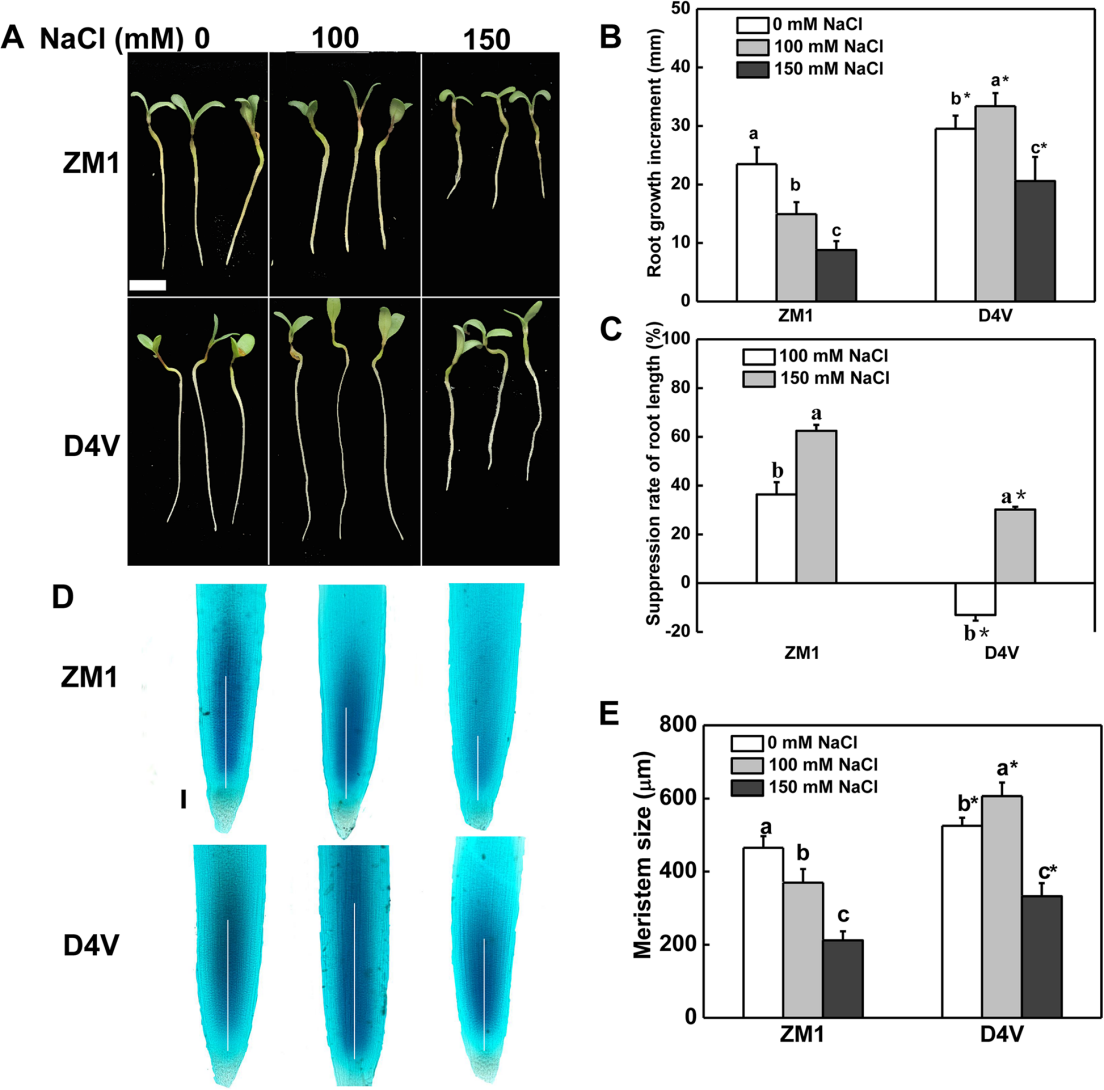
Primary root development of D4V and ZM1 under salt stress. **A** Primary root phenotypes. The bar signifies 0.5 cm. **B** Increase in primary root growth. **C** Primary root growth rate suppression. **D** Micrograph of primary roots. Bar indicates 100 μm; The white lines indicate meristem zone. **E** Meristem size. Three days following treatment, photographs and measurements are conducted. Data values are the means of three biological replicates and standard deviation, with each replicate containing at least ten plants. By Tukey test, lowercase letters indicate a significant difference at *p* < 0.05 between different concentrations of salt stress within the same variety. By t-test, asterisks show a significant difference at *p* < 0.05 between D4V and ZM1 under the same treatment

Furthermore, the primary root development of D4V and ZM1 was studied under progressively increasing NaCl (0, 100, 150 mM). Interestingly, the primary root elongation of ZM1 were inhibited by 36.33% under 100 mM NaCl treatment, while that of D4V showed an 13.08% increase (Fig. 1A and B). Furthermore, NaCl at 150 mM resulted in a 2.3-fold decrease in root increment in ZM1 compared to D4V (Fig. 1A and B). The suppression rate of root elongation was consistent with the results of root growth increment (Fig. 1C).

### Cell cycle-related genes is responsible for the different primary root growth

Microscopic observation of root tips showed that D4V possessed longer root meristem than ZM1 in medium without NaCl (Fig. 1D and E). The meristem size of D4V was increased by 15.4% under 100 mM NaCl, but that of ZM1 was decreased by 20.4%. NaCl at 150 mM resulted in a 36.6% and 54.4% decline in the meristem size of D4V and ZM1, respectively (Fig. 1E). These results were coincident with changes in primary root growth and indicated a physiological relevance between root growth and meristem size in root apical.

**Fig. 2:**
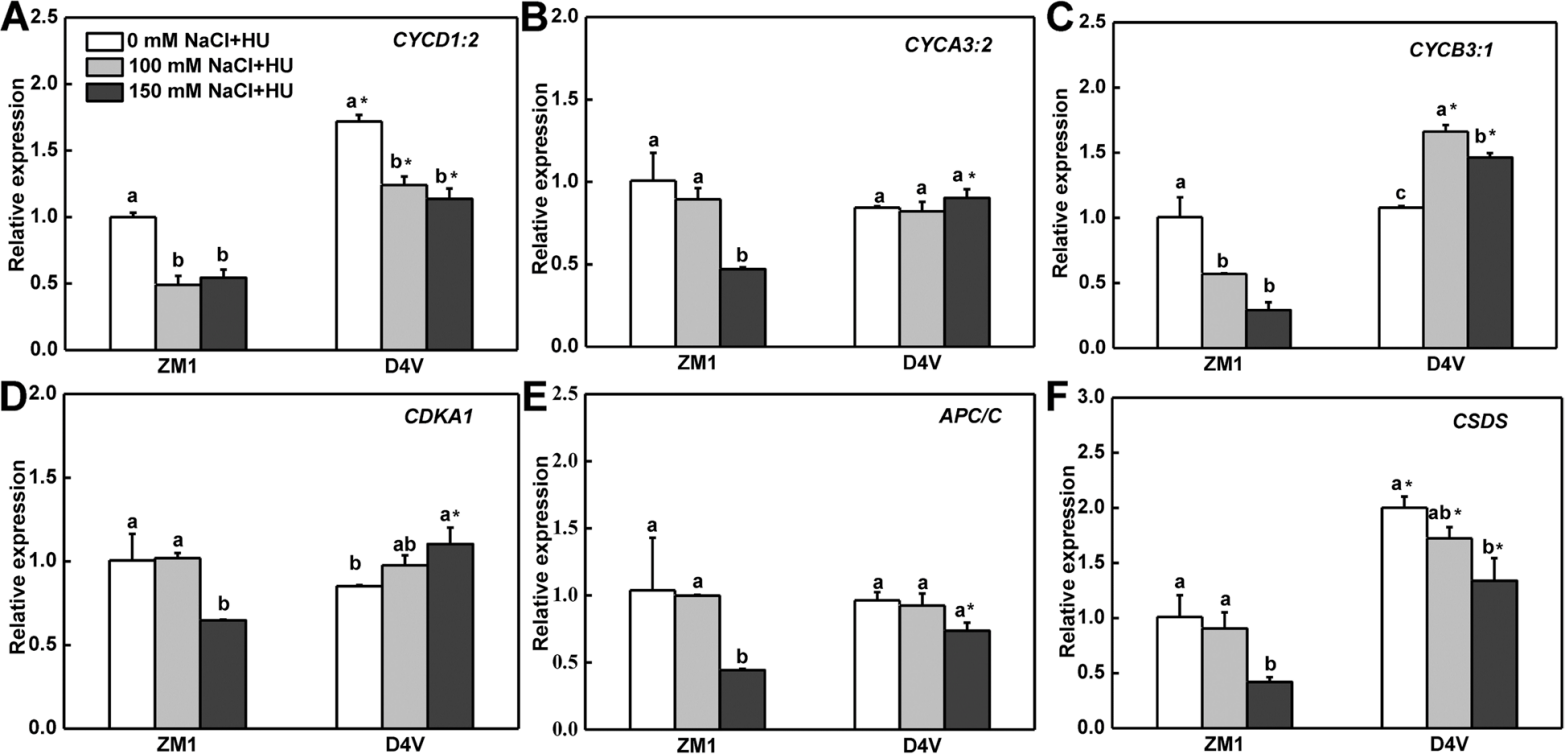
Relative expression of cell cycle related genes in root tips. The data values are means of three biological replicates and standard deviation. By Tukey test, different lowercase letters indicate a significant difference at *p* < 0.05 across different concentrations of salt stress within the same variety. By t-test, asterisks show a significant difference *p* < 0.05 between D4V and ZM1 under the same treatment. 2 mM HU was added to synchronize cell division in the root apical meristem

To further investigate the effect of salt stress on mitosis, the expression of cell cycle-related genes was measured. Under control condition, *CYCD1;2*, a G1 phase gene, showed higher expression level in D4V than ZM1. Furthermore, NaCl at 100 and 150 mM reduced *CYCD1;2* expression arbitrarily, but D4V exhibited higher expression levels of *CYCD1;2* than ZM1 under all conditions (Fig. 2A). *CYCA3;2* showed similar expression level in ZM1 and D4V under normal and 100 mM NaCl conditions, however, it was significantly downregulated in ZM1 but not in D4V by 150 mM NaCl treatment (Fig. 2B). *CYCB3;1*, a G2/M specific gene, was downregulated in ZM1 but increased in D4V by all salt treatments (Fig. 2C). The expression of *CDKA1* was decreased by 36.6% by 150 mM NaCl in ZM1, whereas in D4V, the it was slightly upregulated under the same salt treatment (Fig. 2D). NaCl at 100 mM showed no discernable influence on the expression of *anaphase-promoting complex/cyclosome* (*APC/C*), whereas it was strongly decreased in ZM1 but maintained stable in D4V by 150 mM NaCl (Fig. 2E). *Cyclin SDS* (*CSDS*) is required in protophase and was significantly inhibited by 150 mM NaCl in both ZM1 and D4V, despite the fact that D4V had a higher expression level than ZM1 independent of salt treatment (Fig. 2F). Overall, the expression levels of these cell-cycle related genes were higher in the primary roots of D4V than ZM1, which were positively correlated with relative salt-tolerance of ZM1 and D4V at primary root developmental stage.

### ZM1 was greater in antioxidant capacity and maintaining auxin distribution

**Fig. 3:**
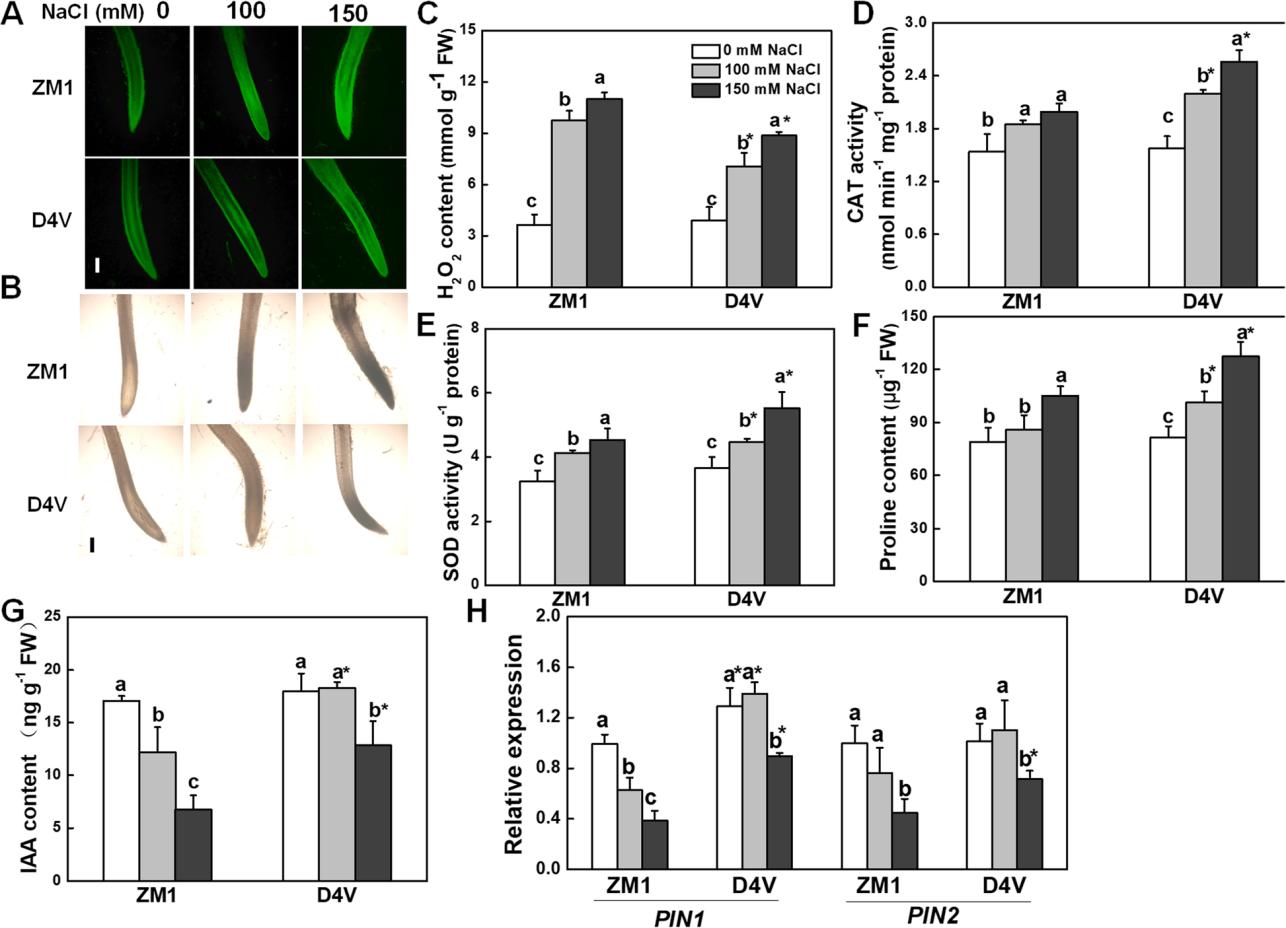
ROS and IAA distribution in root tips of D4V and ZM1 under salt stress. **A** H_2_DCF-DA (**A**) and DAB (**B**) staining for H_2_O_2_. Bar indicates 100 μm; **C** Content of H_2_O_2_. **D** CAT activity; **E** SOD activity; **F** Content of proline; **G** Content of IAA; **H** Relative expression of *PIN1* and *PIN2*. By Tukey test, different lowercase letters indicate a significant difference at *p* < 0.05 across different concentrations of salt stress within the same variety. By t-test, asterisks show a significant difference *p* < 0.05 between D4V and ZM1 under the same treatment. 2 mM HU was added to synchronize cell division in the root apical meristem

ROS accumulation caused by salt stress in root tips are one of the limiting factors of meristem activity. The H_2_O_2_ accumulation and antioxidant potential in primary root of ZM1 and D4V were measured. ZM1 and D4V exhibited a similar level of H_2_O_2_ accumulation under normal condition, indicated by equal intensity of fluorescence and brown coloration, as well as H_2_O_2_ content (Fig. 3A-C). Salt stress triggered H_2_O_2_ in both ZM1 and D4V in a dose-dependent manner, with the former producing more H_2_O_2_ under 100 and 150 mM NaCl treatment (Fig. 3A-C). To further explore whether antioxidant enzymes and proline were related to the differences in H_2_O_2_ generation, activities of the major H_2_O_2_ scavenging enzymes, SOD and CAT, were compared in ZM1 and D4V. As shown in Fig. 3D-F, salt stress induced SOD, CAT and proline in the primary roots of both ZM1 and D4V. However, their induction was significantly more pronounced in the primary roots of D4V compared to that in ZM1 (Fig. 3D and E).

Moreover, content of IAA, the major former of auxin was detected in primary roots. salt stress led to a significant reduction in the distribution of IAA in the root tips of both ZM1 and D4V, with a greater reduction observed in ZM1. Specifically, the distribution of IAA was decreased by 60.3% and 28.5% in ZM1 and D4V, respectively, under 150 mM NaCl treatment (Fig. 3G). Similar to the changes in IAA content, 150 mM NaCl resulted in a significant downregulation of *PIN1* and *PIN2* in both D4V and ZM1, whereas their expression level was higher in D4V compared with ZM1 (Fig. 3H). In addition, the expression of *PIN1* was specifically suppressed in the primary roots of ZM1 upon exposure to 100 mM NaCl, while no such inhibition was observed in D4V. Notably, the expression level of *PIN1* was higher in D4V even under normal condition (Fig. 3H). These findings further evidenced that D4V exhibits greater salt tolerance in primary root growth compared to ZM1, owing to its ability to better maintain ROS and auxin homeostasis during the early developmental stage.

### Effects of salt stress on young seedlings of alfalfa

**Fig. 4:**
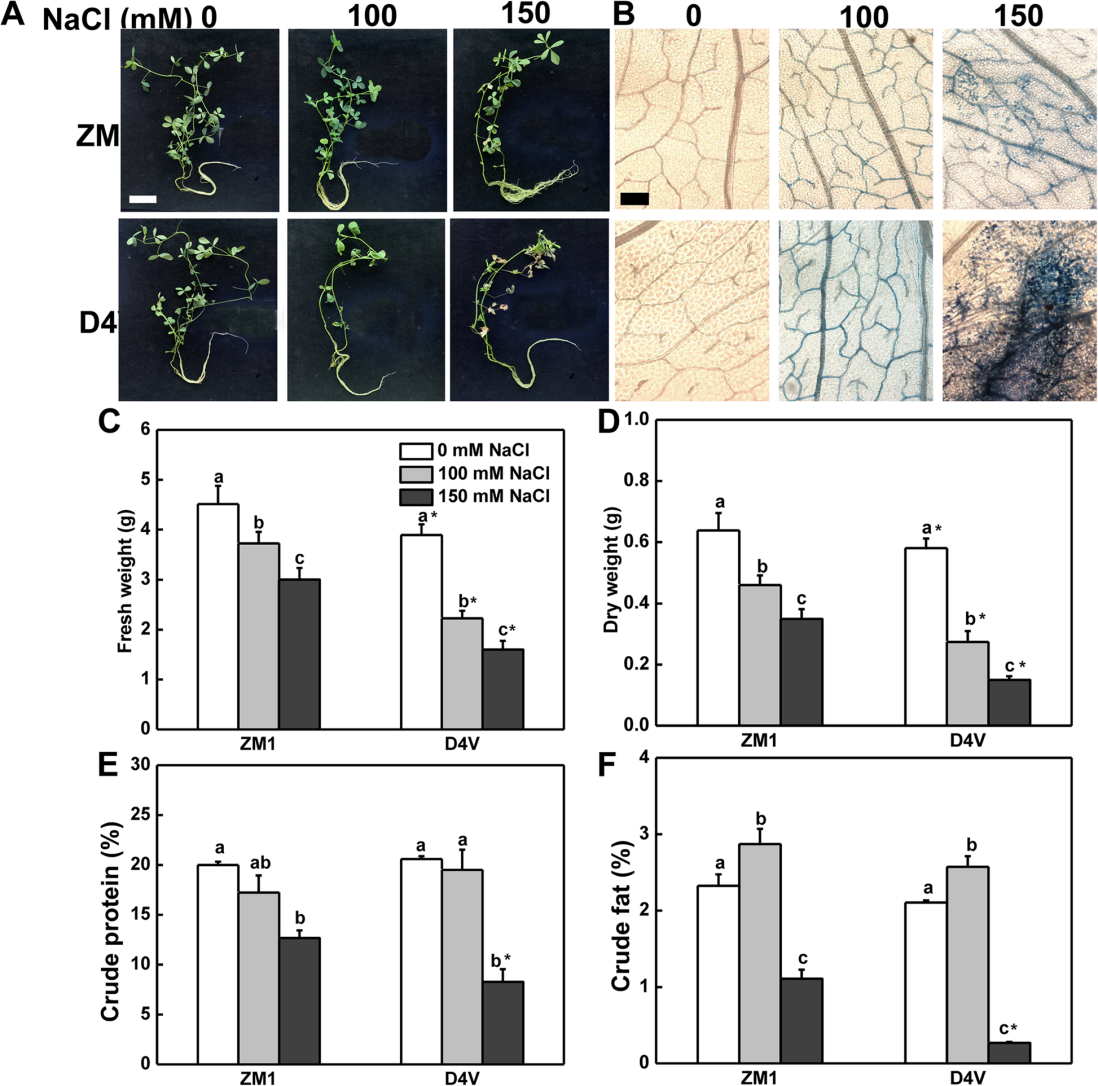
Effects of salt stress on D4V and ZM1 seedlings. **A** D4V and ZM1 seedling phenotypes at varied salt stress concentrations. Bar = 5 cm. **B** Trypan blue staining to show leaf cell death. Bar = 20 μm. **C** Fresh weight. **D** Dry weight. **E** Crude proein; **F** Crude fat. The data are means of at least 8 plants and standard deviation. By Tukey test, different lowercase letters indicate a significant difference at *p* < 0.05 across different concentrations of salt stress within the same variety. By t-test, asterisks show a significant difference *p* < 0.05 between D4V and ZM1 under the same treatment

Salt tolerance of young seedlings of ZM1 and D4V was further analyzed. Intriguingly, D4V, the tolerant variety at early developmental stage, showed a more severe wilting phenotype under both 100 and 150 mM NaCl treatments as compared with ZM1 (Fig. 4A). Salt stress resulted in leaf cell death of D4V and ZM1 as indicated by trypan blue staining in a dose-dependent manner. However, salt induced more severe damage in cell viability in D4V than ZM1 (Fig. 4B). Fresh and dry weight were decreased significantly by salt stress. D4V, on the other hand, was more susceptible to salt-decreased biomass accumulation (Fig. 4C and D). Crude protein and crude fat were used as criterions for forage nutritional quality. As shown in Fig. 4E and F, there was no significant difference between ZM1 and D4V in crude protein and crude fat content under control and 100 mM NaCl treatment conditions. NaCl at 100 mM slightly increased crude fat content in both ZM1 and D4V (Fig. 4F). However, NaCl at 150 mM dramatically decreased the content of crude protein and crude fat indiscriminately, with ZM1 possessing higher forage quality than D4V (Fig. 4E and F). These findings suggested that ZM1 was more resistant to salt stress than D4V at young seedling stage, which contradicted with the performance of their primary root growth at early developmental stage.

**Fig. 5:**
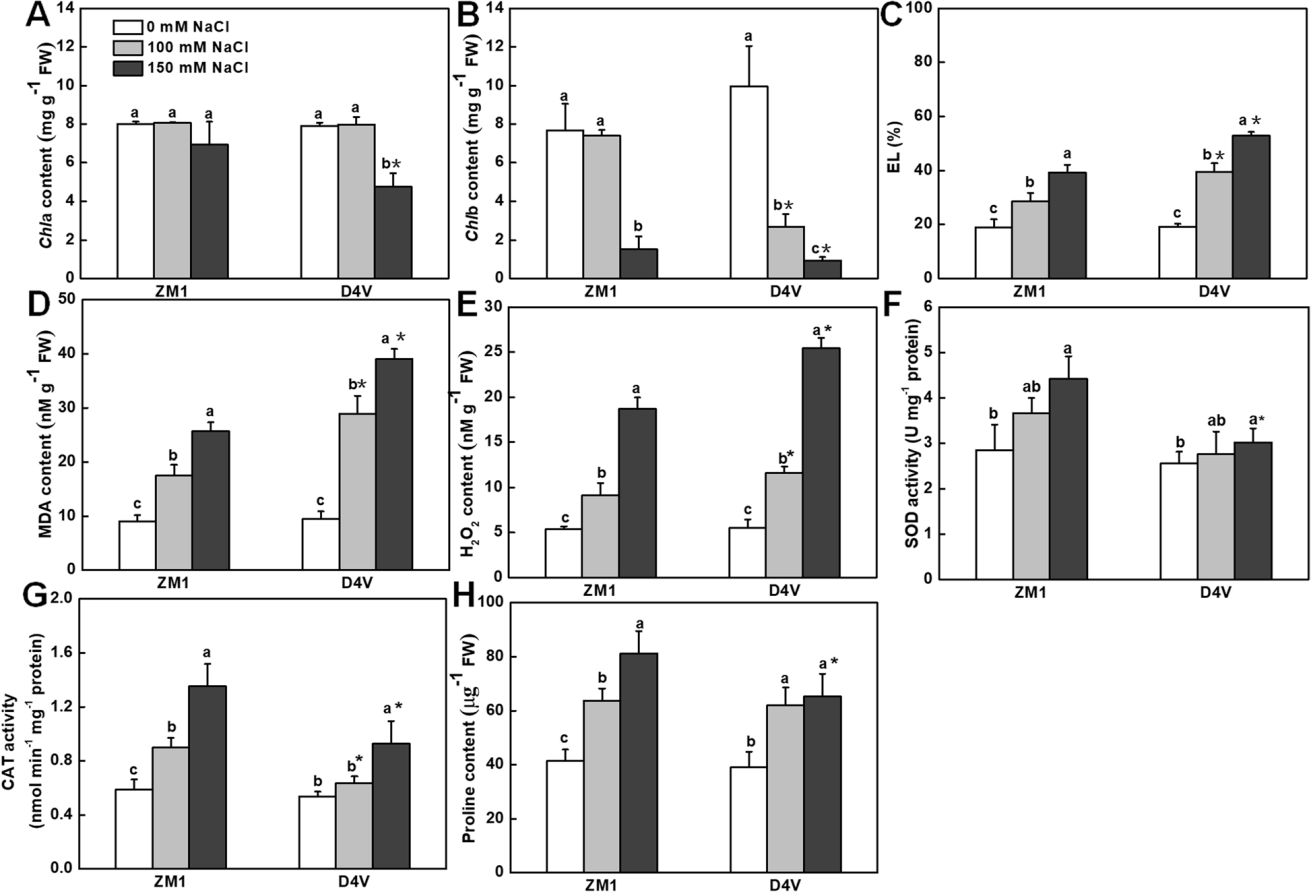
Effects of salt stress on physiological parameters of leaves in D4V and ZM1. **A** Content of *chl* a; **B** Content of *chl* b; **C** EL; **D** Content of MDA. **E** Content of H_2_O_2_; **F** SOD activity; **G** CAT activity; **H** Content of proline. The data values are means of three biological replicates and standard deviation. By Tukey test, different lowercase letters indicate a significant difference at *p* < 0.05 across different concentrations of salt stress within the same variety. By t-test, asterisks show a significant difference *p* < 0.05 between D4V and ZM1 under the same treatment

Consistent with the foregoing phenotype, the negative effects of salt stress on chlorophyll stabilization (Fig. 5A and B), EL (Fig. 5C), and lipid peroxidation (as evidenced by MDA content, Fig. 5D) were more severe in leaves of D4V than ZM1, regardless of the NaCl concentration. Salt stress caused no significant reduction in *chl* a content in ZM1. However, in D4V, treatment with 150 mM NaCl led to a 40% decrease in *chl* a content (Fig. 5A). Different from the change of *chl* a, treatment with 100 mM NaCl reduced the content of *chl* b by 73% in D4V, whereas had no effect on that in ZM1. The content of *chl* b was further reduced under 150 mM NaCl treatment in both ZM1 and D4V, despite its higher content in ZM1 (Fig. 5B). Furthermore, salt stress induced the generation of H_2_O_2_ in leaves of both ZM1 and D4V, whereas the induction was much greater in D4V, which showed a higher level of H_2_O_2_ content in its leaves (Fig. 5E). Paralleled to the less accumulation of H_2_O_2_, ZM1 exhibited significantly higher level of proline content and stronger activity of SOD and CAT under salt stress, compared to D4V (Fig. 5F-H). These findings indicated the greater antioxidant ability in leaves of ZM1 as compared to D4V at young seedling stage.

### Salt-tolerance of young seedlings is associated with Na^+^ tissue-distribution

**Fig. 6:**
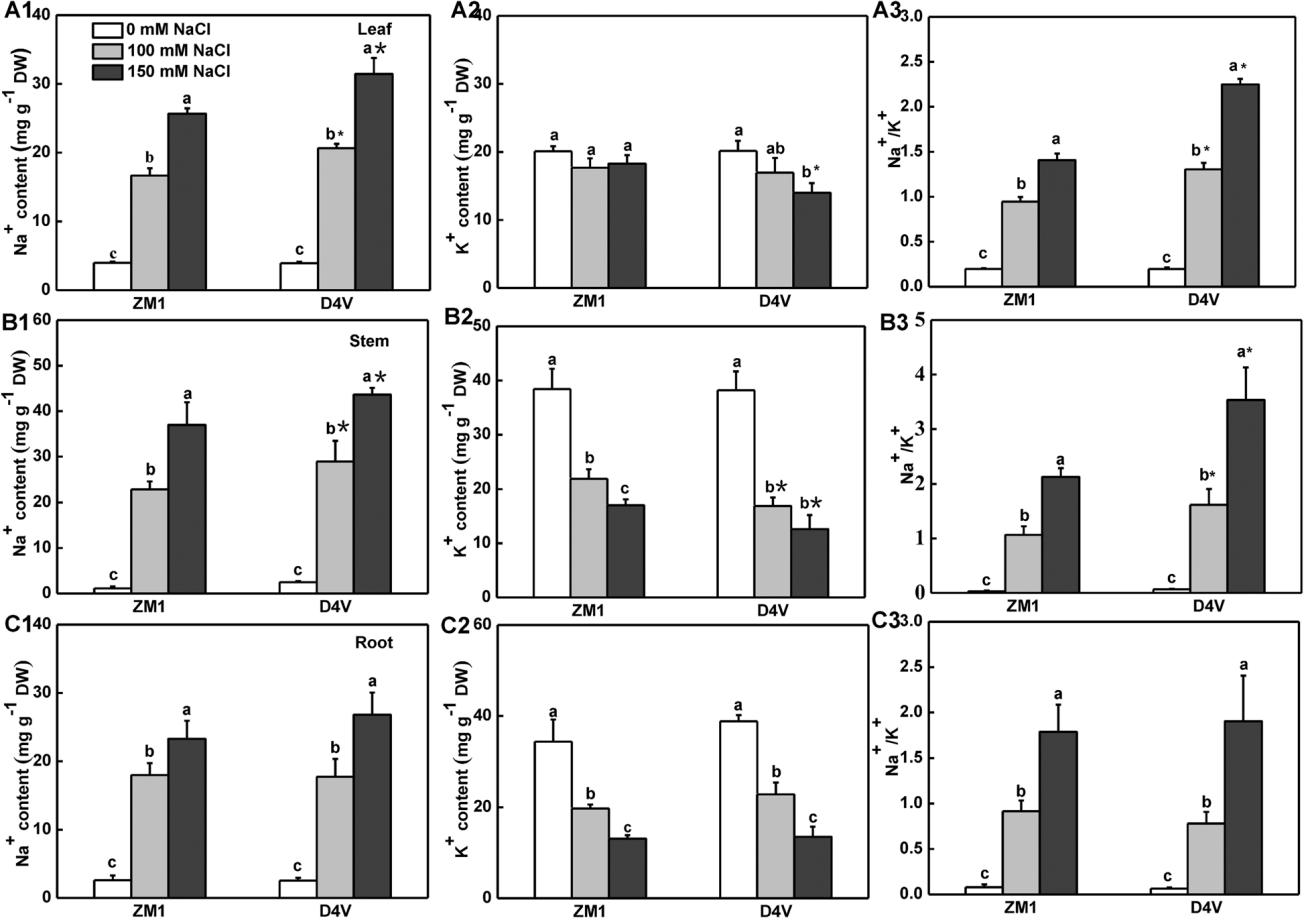
Effects of salt stress on Na^+^, K^+^, and Na^+^/K^+^ accumulation. Leaves, (**A**1-**A**3). Stems **B**1-**B**3. Roots, (**C**1-**C**3). The data values are means and standard deviation (*n* = 3). By Tukey test, different lowercase letters indicate a significant difference at *p* < 0.05 across different concentrations of salt stress within the same variety. By t-test, asterisks show a significant difference *p* < 0.05 between D4V and ZM1 under the same treatment

To further understand the variation in salt tolerance during young seedling stage, we measured the distribution of Na^+^ and K^+^ in leaves, stems and roots. Salt stress resulted in a remarkable increase in the level of Na^+^ in both ZM1 and D4V in a dose-dependent manner (Fig. 6A1, B1 and C1). Under salt stress, D4V accumulated more Na^+^ in the leaves and stems than ZM1 (Fig. 6A1 and B1). Similarly, the K^+^ concentration in leaves (under 150 mM NaCl) and stems (under 100 and 150 mM NaCl) of D4V was lower than that of ZM1 (Fig. 6A2 and B2). As a consequence, the Na^+^/K^+^ in leaves and stems of D4V was 1.4–1.6 times higher than that of ZM1 under salt conditions (Fig. 6A3 and B3). Furthermore, we found no change in Na^+^ and K^+^ concentration or Na^+^/K^+^ in roots between the two kinds (Fig. 6C1, C2 and C3). Therefore, the overall Na^+^ accumulation and Na^+^/K^+^ ratios in the leaves and stems of ZM1 were lower compared to D4V under salt stress.

**Fig. 7:**
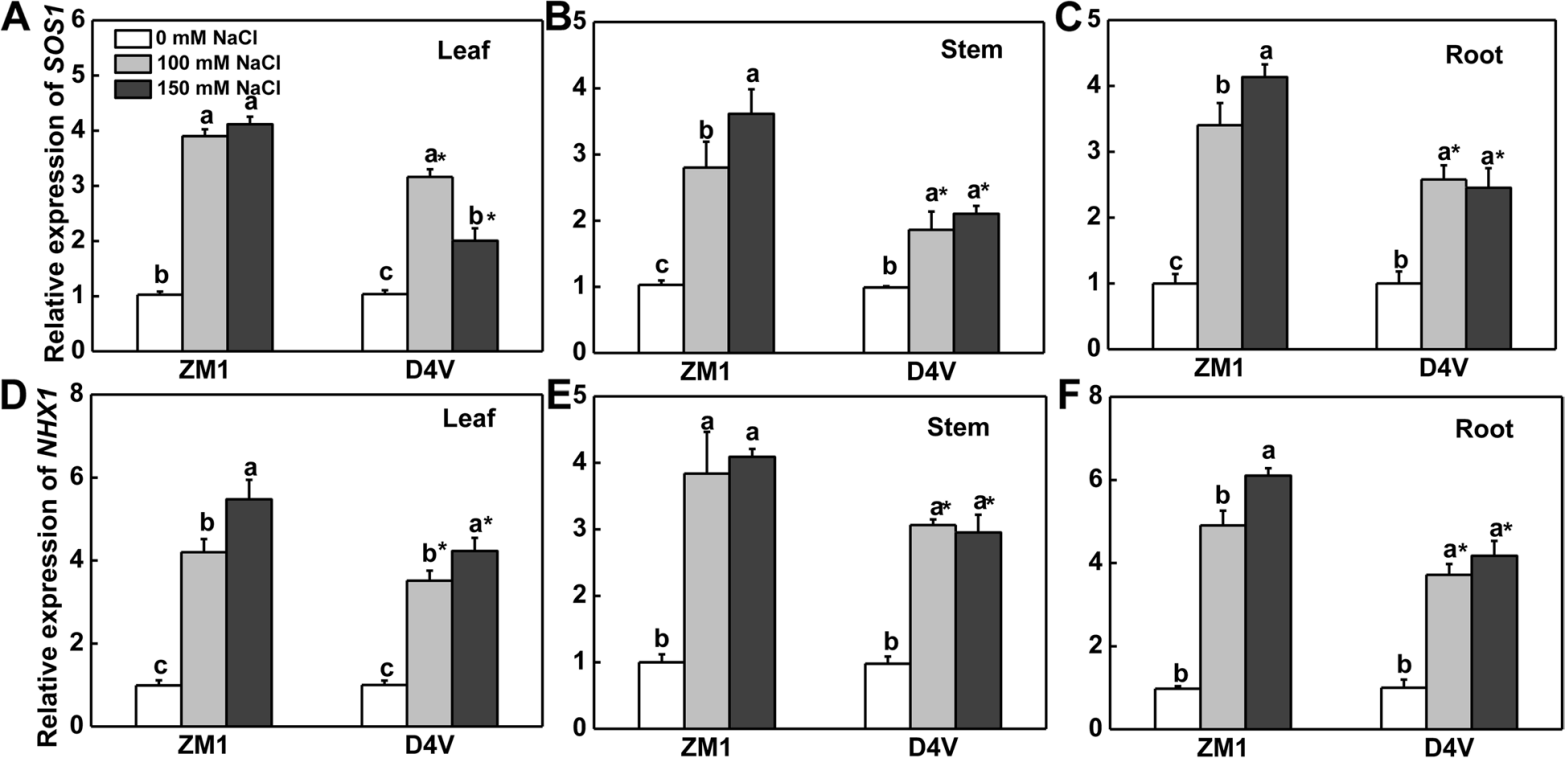
Relative expression of genes encoding ion transporters. **A**-**C** Relative expression of *SOS1* in leaf (**A**), stem (**B**) and root (**C**). **D**-**F** Relative expression of *NHX1* in leaf (**D**), stem (**E**) and root (**F**). Data are means of three biological replicates and standard deviation. By Tukey test, different lowercase letters indicate a significant difference at *p* < 0.05 across different concentrations of salt stress within the same variety. By t-test, asterisks show a significant difference *p* < 0.05 between D4V and ZM1 under the same treatment

**Fig. 8:**
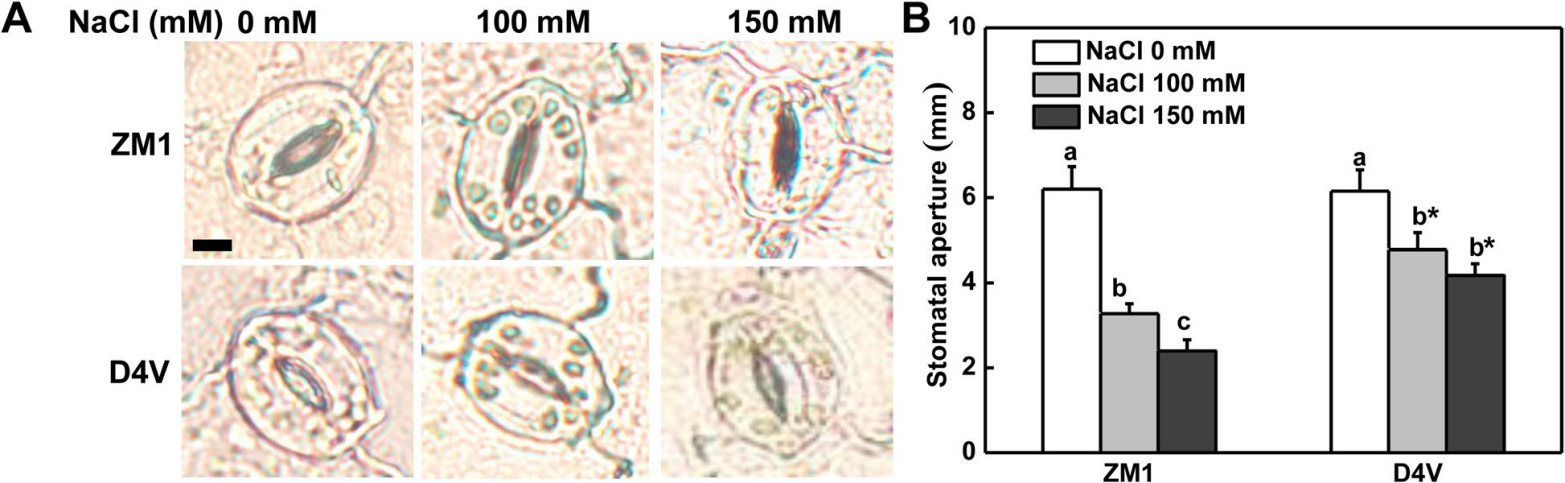
The influence of salt stress on stomatal aperture. The bar in A represent 10 μm. The results represent the means of three biological replicates and standard deviation, with each replication being an average of stomata in a microscope field including at least 20 stomata. By Tukey test, different lowercase letters indicate a significant difference at *p* < 0.05 across different concentrations of salt stress within the same variety. By t-test, asterisks show a significant difference *p* < 0.05 between D4V and ZM1 under the same treatment

Salt stress dramatically upregulated the expression of genes encoding plasma membrane (*SOS1*) and tonoplast (*NHX1*) ion transporters in leaves, stems and roots of ZM1 and D4V indiscriminately (Fig. 7). With the exception of *SOS1* in leaves and *NHK1* in stems, salt stress induced these genes in a dose-dependent manner in ZM1. However, 150 mM NaCl could not resulted in further upregulation of these genes in D4V except for *NHX1* in leaves (Fig. 7). Crucially, ZM1 possessed higher transcription level of both *SOS1* and *NHX1* in leaves, stems and roots than D4V, concomitant with the lower accumulation of Na^+^ and higher concentration of K^+^ of the whole plants (Fig. 7). Besides ion transporters, microscopic analysis showed that salt stress caused a decrease in stomatal aperture of both ZM1 and D4V, as predicted (Fig. 8A and B). Surprisingly, ZM1 exhibited a greater reduction in stomatal aperture when subjected to 100 and 150 mM NaCl (Fig. 8). As a result, the lower Na^+^ buildup in ZM1 leaves and stems was ascribed in the higher expression of ion transporter genes and greater sensitivity of stomatal closure under salt stress, which guaranteed the greater salt-tolerance in ZM1 at young seedling stage.

## Discussion

The primary root, as a fundamental component of the root system, governs plant seedling growth and development [31]. Furthermore, the primary root is the first organ to be exposed to and affected by salt stress [5]. Previous research has found that salt stress inhibited primary root development, while tolerance-enhanced regimes mitigated this type of inhibition [32]. Consistently, a significant suppression of primary root growth was observed in both D4V and ZM1 in our study when they were exposed to 150 mM NaCl. Notably, the inhibition was more severe in ZM1 compared to D4V. In rice, the salt-tolerant varieties showed longer primary root under salt stress [33]. Thus, it was suggested that D4V had a greater salt tolerance than ZM1 at early primary root growth stage. Furthermore, the reaction of D4V to moderate salt stress separated it from ZM1. NaCl at 100 mM resulted in a slight increase in primary root growth in D4V but a significant decrease in ZM1 (Fig. 1). The effects of salt stress on primary root growth were closely associated with the changes in phytohormones such as ABA. ABA was reported to promote primary root growth at low concentration and inhibit it at high concentration [9, 34]. Therefore, the different response of D4V and ZM1 under 100 mM NaCl seems to be related to the different in ABA production or sensitivity.

Plants employ a variety of strategies to maintain primary root development under salt stress, the majority of which are alteration of cell division or elongation in root apical [35, 36]. Previous research found that salt stress slowed primary root development by impairing cell division in root apical meristem [8, 37]. Although the salt-tolerant alfalfa variety showed a less decrease in primary root length than the sensitive one under salt stress [38], the underlying molecular mechanism is poorly understood. Meristem activity is sustained by the maintenance of cell cycle progression, which is regulated by *CDKs*, *CYCs* and other regulatory genes [39]. Thymidine analogue EdU labeling of active replicated cells revealed that increasing salt stress inhibited replicating frequency [40]. Salt stress was reported to consistently inhibit the expression of *CYCA*, *CYCB*, and *CDK* genes in Arabidopsis [10, 12]. Agreeingly, all tested cell cycle associated genes in this study were shown to be suppressed in the root tips of ZM1 by salt stress. However, D4V showed higher expression level of these genes under salt stress and even normal condition (Fig. 2). An intriguing expression pattern was found in *CYCB3;1*, which was dramatically suppressed in ZM1, whereas slightly induced in D4V by salt stress. Similarly, a B-type cycling gene *CYCB1* was reported to be induced by 100 and 200 mM NaCl in monocotyledonous *Brachypodium distachyon* [40], whereas the homologous was gradually suppressed from 50 mM NaCl onwards in dicotyledonous Arabidopsis [41, 42]. The opposite expression pattern may be related to plant species differing in salt tolerance. When deciphering our data according to this point, the higher expression levels of cell cycle related genes endow the greater salt tolerance in primary root of D4V. These differentially expressed genes may be targets for salt-tolerant alfalfa breeding.

Generation of ROS under salinity conditions imposes oxidative stress on primary roots and severely limits it growth. The study of the *iar* mutant in Arabidopsis showed that the hypersensitivity of primary root development to salt stress in this mutant was caused by the higher accumulation of ROS, due to ineffective induction of ROS scavenging system in response to salt stress [8]. Consistently, the more significant inhibitory effect of salt stress on primary root development of ZM1 was accompanied by overaccumulation of H_2_O_2_ in root tips and weaker induction of antioxidants (Fig. 3A-F). Moreover, local distribution of auxin mediated by polar auxin transport is a key regulator of primary root growth and meristem activity [43]. Overexpression of a auxin receptor, *AFB3* in Arabidopsis enhanced the salt tolerance by rescuing the salt-inhibited primary root elongation [44]. In the *iar* mutant, a weaker DR5-GUS staining and a reduced root meristem size were observed under salt stress [8]. Paralleled to the previous results, the longer primary root and greater root meristem activity in D4V were along with the higher distribution of IAA in root tips and expression level of *PIN* genes (Fig. 3 G and H). This suggests that IAA may play a role in improving the salt tolerance of D4V at early primary root developmental stage.

At young seedling stage, both 100 and 150 mM NaCl generated cell death and membrane damage in ZM1 and D4V, divergent from the inducing effect of 100 mM NaCl on D4V primary root growth. This result supported prior findings that plants at the immature seedling stage were more susceptible to salt stress [45, 46]. Remarkably, young seedlings of ZM1 were more salt-tolerant than D4V, exhibiting milder cell death, growth retardation, membrane peroxidation and chlorophyll degradation (Figs. 4 and 5), supporting the notion that ZM1 was a salt tolerant variety at the seedling stage [47]. Salt stress response is an energy-consuming process, resulting in growth reduction and organic degradation [3]. Maintaining higher yield and nutritional quality under salt condition are major aims for forage breeding [48]. ZM1 exhibited a greater ability to alleviate the negative effects of salt stress on plant biomass and nutrient content compared to D4V, indicating that the stronger salt tolerance of alfalfa protected biochemicals from degradation under salt condition. Higher concentration of crude protein under 150 mM NaCl might be attributed to superior nitrogen uptake, because overexpression of H^+^-PPase enhanced nitrogen use efficiency [49] and this gene also improved salt tolerance of alfalfa [50]. More importantly, the relative salt tolerance of the two tested varieties was opposite at different developmental stages. In fact, the variations in relative order of salt resistance for different varieties during the course of growth and development have been documented for decades [29, 51–53]. In rice, some salt-resistant varieties during germination were found to be salt-sensitive at young seedling or tillering stages. The relative order of salt tolerance changed due to the variation in salt tolerance of one specific genotype at different stages, and this kind variation exhibited different patterns among genotypes [29]. We postulated that the different behaves of salt tolerance at different stages was a result of phenological development. In order to adapt to the periodic changes of their habitat environment including saline changes, different varieties evolved different growth, development, and stress response patterns. Furthermore, genomic investigation revealed that several genes implicated in salt tolerance have a stage-specific expression pattern [54, 55], which might be a plausible mechanism controlling salt tolerance in a stage-dependent way.

Ionic toxicity and oxidative damage are main components of salt stress. Generally, enhancing in antioxidant capacity can improve salt tolerance. Overexpression of *CuZnSOD* conferred salt tolerance of salt sensitive sweet potato [56]. Researches on association between antioxidant metabolism and alkali-salt tolerance of switchgrass revealed that varieties with higher CAT activity possessed better alkali-salt tolerance [57]. Therefore, the increased antioxidant enzymes in leaves of ZM1 are facilitated to its greater salt tolerance (Fig. 5C-H). Decreased Na^+^ buildup in leaves is a conserved process that facilitates salt tolerance in a wide variety of plants. A comparison of Na^+^ tissue distribution in different wheat types revealed that Na^+^ sequestration in roots had a role in distinguishing salt stress tolerance [58]. Generally, the salt-tolerant types tended to accumulate less Na^+^ in leaves than the sensitive ones when comparing different genotypes of one species [59, 60]. Further kinetic examination of Na^+^ transit in different tissues emphasized that salt-tolerant barley maintained more Na^+^ in roots by avoiding Na^+^ uploading in xylem sap, when compared to salt-sensitive rice [61]. In line with prior researches, the salt-tolerant ZM1 in the current test had decreased Na^+^ in leaf and stem than D4V (Fig. 6A1 and B1). Considering the fact that there was no significant difference in roots Na^+^ accumulation between the two varieties, ZM1 was better in reducing Na^+^ uptake and leaf-distribution. The amount of Na^+^ accumulated in leaves was determined not only by the Na^+^ concentration in xylem sap controlled by ion transporters but also by the transpiration pull regulated by stomata movement [3]. *SOS* and *NHX* gene families are major regulators of cytoplasmic Na^+^ and K^+^ concentration and Na^+^/K^+^ ratio. Among the SOS cascade, *SOS1* was reported to function in excluding Na^+^ from roots and retrieving it from the xylem, thereby preventing the accumulation of Na^+^ in leaves [21]. The transcription level of *SOS1* was higher in ZM1 than D4V under salt stress (Fig. 7A-C), deciphering the lower concentration of Na^+^ in leaves and stems of ZM1. *NHX1* had equal affinity to Na^+^ and K^+^, which helps maintain K^+^ homeostasis and an appropriate Na^+^/K^+^ ratio during salt exposure [48]. ZM1 kept a higher level of K^+^ and a lower Na^+^/K^+^ ratio in stems and leaves (Fig. 6A1-A3, B1-B3), which might be linked with the stronger induction of *NHX1* expression (Fig. 7D-F). Additionally, Stomata shrank as a result of salt stress [62]. Different studies reached contradictory conclusions about the role of stomata closure in salt stress response. On the one hand, salt-induced stomata closure reduced water loss and Na^+^ upward transfer [63, 64]. Stomata closure, on the other hand, impeded carbon absorption and lowered photosynthesis, which was detrimental to salt tolerance [65]. In our experiment, the greater salt sensitivity of D4V was accompanied by increased stomata conductance compared to ZM1 (Fig. 8). Moreover, impaired K^+^ homeostasis decreased the sensitivity of stomata closure to salinity [66]. From this point of view, delayed stomata closure in leaves of D4V might be a result of ion imbalance. Therefore, ion transport and stomata movement synergistically regulated the ion homeostasis through positive feedback.

## Conclusion

**Fig. 9:**
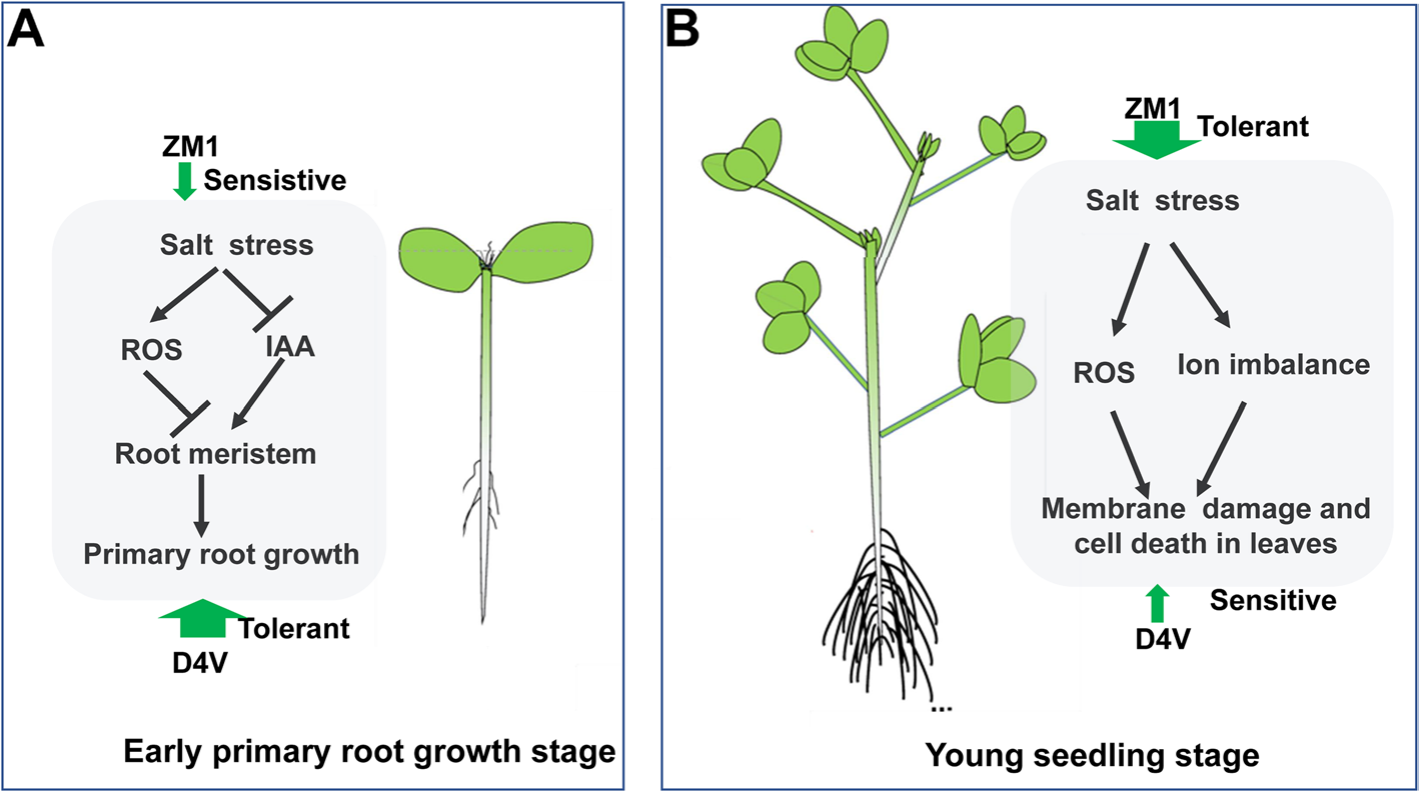
A model describing the contrasting reactions to salt stress of two alfalfa types, ZM1 and D4V, at the primary root development and young seedling stages. The thick and thin arrows indicate salt tolerant and sensitive, respectively

In this study, we aimed at the specific physiological responses of two alfalfa varieties, ZM1 and D4V, during the early primary root growth and young seedling stages. The relative order of salt tolerance varied at different developmental stages. Firstly, physiological and molecular evidence supports that D4V was more salt-tolerant than ZM1 during the early primary root growth due to the advantages in maintaining meristem size. This was achieved through maintaining higher expression level of cell cycle-related genes, greater antioxidant capacity and more efficient auxin transport (Fig. 9A). Nevertheless, ZM1 obtained a stronger induction of antioxidant systems in leaves and was more effective at inhibiting Na^+^ transfer from root to leaf. This was due to the advantages in activating ion transporters and triggering stomatal closure, ultimately led to a greater salt tolerance in ZM1 than D4V at young seedling stages (Fig. 9B). Goals of breeding for salt-tolerant alfalfa is to enhance the salt tolerance throughout the growing season. We have provided compelling mechanisms for alfalfa varieties to cope with salt stress at specific developmental stages. Further researches are necessary to reveal how alfalfa regulates stage-specifically physiological reactions at cell and molecular levels, thus assisting plant salt-tolerant breeding.

## Materials and methods

### Plant materials and growth conditions

In this study, eight varieties of alfalfa were used as plant materials: ZM1, D4V, Algonquin, Yanbao, Zhongmu No. 3 (ZM3), WL-SQT, Biaoba, and Longmu 801. Plant materials for all treatments were cultivated in an artificial climate chamber (Saifu, Ningbo, China, ZRY-YY1000) at 28 °C (day)/25 °C (night) for 14 h (light)/10 h (dark). The photosynthetic photon flux density (PPFD) was kept at 600 μmol m^−2^ s^−1^ from fluorescent tubes. The relative humidity was kept constant at 70%.

### Experiment design

- (i) To assess the various responses of seed germination and primary root growth to salt stress, healthy and full seeds of eight alfalfa types were manually scarified, sterilized with 10% NaClO, and germinated in petri dishes containing filter papers soaked by gradient increasing NaCl (0, 25, 50, 100, 150 mM). Each treatment has three replicates and each replicate has 100 seeds. The germination percentage was determined two days after treatment. Based on the result of germination percentage that 150 mM NaCl significantly inhibited seed germination of all varieties, relative salt tolerance of the tested varieties was estimated under 150 mM NaCl. Briefly, the sterilized seeds with 1 cm radicles germinated under control conditions were chosen and placed in 1/2 MS medium with or without 150 mM NaCl. The starting position of root tips was identified and the increase in root growth was detected three days after treatment.
- (ii) To assess the response of root elongation to different degree of salt stress, D4V and ZM1 were selected as salt-tolerant and sensitive varieties at early developmental stage respectively on the basis of (i). Seeds with radicles at 1 cm of D4V and ZM1 were treated with 0, 100 and 150 mM NaCl. Each treatment has three replicates. Three days following treatment, the root length increase was measured. To examine the expression of cell cycle-related genes, 2 mM hydroxyurea (Hu, Sigma-Aldrich) was added to the medium to synchronize the cell cycle. Three days following treatment, root tips were collected.
- (iii) To compare the seedling performance of D4V and ZM1, seedlings were cultivated in a peat and vermiculite combination (v/v, 2:1). After three weeks, seedlings were transplanted into a 1/2 Hoagland nutrition. After adaptable growth for two weeks, at least eight healthy alfalfa plants for each variety were salt treated with 0, 100 and 150 mM NaCl solution. In detail, NaCl was gradually increased at the speed of 50 mM per day from the first day of treatment before reaching to the final concentration. Samples for genes expression analysis was collected after salt treatment for 24 h. Cell death, EL, MDA, chlorophyll and ion content were all measured 7 d after treatment.

### Measurements

#### Root meristem size

Root tips were fixed in acetic acid/ acetaldehyde (v/v, 3:1) for 24 h and then stained by 0.1% methylene blue for 5 min. after washed for 3–5 times used deionized water, the root tips were decolorized in saturated chloral hydrate solution containing 10% glycerol. The root tips were immobilized onto a glass slide by decolorizing solution above mentioned and covered with coverslips. Root meristem was visualized and photographed at a magnification of 100 × using a microscope (Zeiss, Germany). Root meristem size (from quiescent centre to elongation zone) was measured using image J software. There were at least 10 roots were measured for each treatment.

#### Histochemical observation of H_2_O_2_ in root tips

To visualize the distribution of hydrogen peroxide H_2_O_2_ in root tips, we performed DAB (3,3'-diaminobenzidine, Sigma) staining. Briefly, fresh root tips were incubated in 1 mg/mL DAB solution (pH 3.8) for 30 min in the dark. The samples were then decolorized by boiling in ethanol/acetic acid (3:1) until the background was cleared. For H_2_DCF-DA (2',7'-dichlorodihydrofluorescein diacetate, Sigma) staining, fresh root tips were incubated in 10 µM H_2_DCF-DA solution for 30 min at 37 °C in the dark. The samples were then washed three times with phosphate-buffered saline (PBS) and observed under a fluorescence microscope. Both DAB and H_2_DCF-DA staining were performed on at least ten biological replicates, and representative images were obtained.

#### Chlorophyll content

Fresh leaves were cut into 0.5 cm segments and immediately immersed in 10 mL dimethyl sulfoxide for 2 d in the dark. The absorbance of the extracting solution at 663 nm and 645 nm was measured using an ultraviolet spectrophotometer to determine the amount of chlorophyll a and b. Each treatment received three biological replicates.

#### EL and MDA

Fresh leaves (0.2 g) were moistened three times with distilled water and sliced into 0.5 cm strips to measure EL. Leaf segments were immediately inserted in tubes with 25 mL distilled water and shaken at room temperature for 24 h at 200 rpm. A conductivity mete (JENCO-3173, Jenco Instruments, Inc., San Diego, CA, USA) was used to calculate the first EL (EC1). The samples were then boiled for 20 min at 95 °C. After the solution had cooled to room temperature, the maximum of EL (EC2) was observed. The EL was determined using the formula: EL (percent) = EC1/EC2100. Each treatment received three biological replicates.

Frozen leaves (0.2 g) were ground into powder and homogenized in extraction buffer (150 mM PBS at PH = 7.0) to determine MDA concentration. The homogenate was centrifuged at 12000 g for 20 min, and the supernatant was mixed with 0.5% 2-thiobarbituric acid and 20% trichloroacetic acid. The reaction mixture was then heated at 95 °C for 30 min and centrifuged at 12000 rpm for 20 min after cooling to ambient temperature. The absorbance was measured at 600, 532, and 450 nm respectively. The final MDA content was estimated using the difference between OD_532_ and OD_600_. The OD_450_ was employed to remove the effects of sugar and aldehyde. Each treatment received three biological replicates.

#### Trypan blue staining

Leaves were vacuumed into a prewarmed staining buffer (10 mL phenol, 10 mL glycerol, 10 mL lactic acid, 10 mg trypan blue mixed in 10 mL distilled water), cooked at 95 °C for 10 min, and then shaken overnight. To destain the leaves, they were rinsed with chloral hydrate until they became translucent. A microscope was used to view and photograph cells (Zeiss, Germany). For each treatment, at least ten leaves were discolored.

#### Forage quality

Crude protein was determined based on Kjeldahl method (AOAC, 1999). Crude fat was extracted by petroleum ether on Soxherm apparatus according to Soxhlet method (AOAC, 1990).

#### H_2_O_2_ and proline content, and antioxidant enzymes activity

For H_2_O_2_ content detection, 0.1 g fresh samples were homogenized in 1 M HClO4 and centrifuged at 6000 g for 10 min. The supernatant was adjusted to a pH of 6.0–7.0 using 4 M KOH. Equal volumes of the supernatant and reaction buffer, containing 100 mM potassium acetate (pH 4.4) and 1 mM 2,2'-hydra-bis (3-ethylbenzothiazolin-6-sulfonic acid), were mixed. The difference in absorbance at 412 nm after adding POD enzyme and no POD enzyme was used to determine the H_2_O_2_ content. The exact content of H_2_O_2_ was calculated according to a standard curve of a H_2_O_2_ concentration gradient.

For content detection of proline, fresh leaf samples (0.1 g) were homogenized in 10 mL of 3% aqueous sulfosalicylic acid, and the homogenate was centrifuged at 10,000 g for 10 min. The supernatant (2 mL) was mixed with 2 mL of acid ninhydrin and 2 mL of glacial acetic acid. After boiled for 1 h and cooled to room temperature, the chromophore was extracted with 4 mL of toluene. The absorbance of the organic layer was measured at 520 nm using a spectrophotometer. Proline content was calculated based on a standard curve a proline concentration gradient.

To detect the activity of antioxidant enzymes, root samples (0.1 g) were homogenized with 2 mL of ice-cold extraction buffer (50 mM phosphate buffer (PBS, pH 7.8), containing 0.2 mM EDTA, 2 mM L-ascorbic acid, and 2% (w/v) polyvinylpolypyrrolidone). The homogenates were centrifuged at 13000 g for 20 min, and the supernatants were used for the determination of enzyme activity. Peroxidase activity was indicated by the oxidation of guaiacol. Catalase (CAT) activity was measured by the decomposition rate of H_2_O_2_.

#### IAA content detection

Frozen root samples of 0.1 g were homogenized in 1 mL of ethyl acetate, which had been spiked with D6-IAA (C/D/N Isotopes Inc, Canada) as internal standard with a final concentration of 100 ng mL^−1^. After shaking extraction overnight at 4 ℃, the tubes were centrifuged at 13000 rpm for 10 min at 4 ℃. The pellet was re-extracted with 1 mL of ethyl acetate. Both supernatants were evaporated to dryness under N_2_ and the residues were resuspended with 70% methanol (v/v). The filtered supernatants were then analyzed using LS/MS–MS. A negative electrospray ionization mode was used for detection.

#### Gene expression

Total RNA was extracted using an RNAprep pure Plant Kit (TIANGEN, Beijing, China) according to the manufacturer's instructions and quantified using a UV spectrophotometer NanoDropTM (Thermo Fisher Scientific, Lenexa, KS, USA). The first-strand cDNA was synthesized from RNA (500 ng) using a ReverTraAce qPCR RT Kit with genome-DNA-removing enzyme (Toyobo, Osaka, Japan). The qRT-PCR tests were run using a Roche Light Cycler480 detection system with SYBR Super Mix (Takara, RR420A, Shika, Japan). Supplementary Table S1 showed specific primers for target genes. As an internal reference, the alfalfa housekeeping gene *Actin* was employed. The delta-delta Ct technique was used to determine the relative expression of target genes. Each treatment received three biological replicates.

#### Ion content detection

All samples were fixed at 105 °C for 30 min to deactivate enzymes before drying at 75 °C for 48 h. Dried samples (0.1 g) were digested for 12 h with 5 mL HNO3 (69 percent, v/v) before the crude extraction solution was transparentized with H_2_O_2_. The solution was filtered with quality filter paper and diluted with deionized distilled water. An atomic absorption spectrometer was used to measure the Na^+^ and K^+^ content (A6300; Shimadzu, Kyoto, Japan). Each treatment received three biological replicates.

#### Stomata aperture

With a fine-tipped tweezer, the abaxial epidermis of leaves was extracted and floated in a MES solution containing 10 mM KCl. A light microscope (Zeiss, Germany) was used for microscopy with a magnification of 40. Image J was used to measure at least ten stomata.

### Statistical analysis

One-way ANOVA was conducted to analyze the variance of all data by SPSS statistics 20 software. Tukey test was applied to compare the difference of the same variety among different NaCl treatments. T-test was used in pairwise comparison of the two varieties under the same conditions.

## Supplementary Information

**Additional file 1. **

**Additional file 2:** **Table S1.** Primers used for qRT-PCR analysis.

## Data Availability

The datasets used and/or analyzed during the current study are available from the corresponding author on reasonable request.

## Abbreviations

ZM1: Zhongmu No.1
ZM3: Zhongmu No.3
Chl *a*: Chlorophyll a
Chl *b*: Chlorophyll b
MDA: Malondialdehyde
EL: Relative electrolytic leakage
ROS: Reactive oxygen species
CYC: Cyclin
CDK: Cyclin dependent kinase
APC/C: Anaphase-promoting complex/cyclosome
CSDS: Cyclin SDS
HU: Hydroxyurea
SOS1: Salt overly sensitive1
HKT: High affinity Na^+^/K^+^-permeable transporter
NHX: Na^+^/H^+^ exchanger
