# Supplementary material for: Involvement of cell cycle and ion transferring in the salt stress responses of alfalfa varieties at different development stages
Source: BMC Plant Biol. 2023 Jun 27;23:343. doi: 10.1186/s12870-023-04335-3 (PMC10294350; doi:10.1186/s12870-023-04335-3)
Supplement: Supplementary file 2 — Additional file 2: Table S1. Primers used for qRT-PCR analysis. [file 12870_2023_4335_MOESM2_ESM.docx]

| Genes | Forward primers (5’-3’) | Reverse primers (5’-3’) |
| --- | --- | --- |
| *CYCD1;2* | TCTCGCTCTCTCGAAGCCAA | AACGGAGAGGTACGCCGTTA |
| *CYCA3;2* | GGTCTAGAGGGTGTTGATGCT | ACTGATGCAGCCACCATAGA |
| *CYCB3;1* | TGAATCGCGTCCAACTGTGG | GCTGCGCCAAATTCTCTTGC |
| *CDKB2* | CCAAGGTGGGAGCCTCAGAA | TCAAGTGCTGCTTTGGCAGA |
| *APC/C* | TTCCTGGGTGCCACTTACCA | TTCTCGAACCACCACACTGC |
| *CSDS* | GGGTCACGTGCCTTGCATTA | CCACTCCATGCTCTCCATGC |
| *SOS1* | CAGGGATATTGCATGCTGCG | TGGCCAGGGATGTCTACCAT |
| *NHX1* | ACTTCTTGGAAGAGCAGCGT | AGTTGAGTATGCCCCGACAC |
| *PIN1* | TCATGTTGCCGTTGTTCAGG | CGCCATTTCCACTTAGCCAA |
| *PIN2* | ACTGTCTGCTGGTCTTGGAA | GGAAGTGCAGCCTGAACAAT |
| *Actin* | CCGACCTCGTCATACTGGTG | TCTTCAGGAGCAACACGCAA |

**Table S1.** Primers used for qRT-PCR analysis
